# Effects of Oxytocin on Facial Expression and Identity Working Memory Are Found in Females but Not Males

**DOI:** 10.3389/fnins.2018.00205

**Published:** 2018-04-17

**Authors:** Tong Yue, Caizhen Yue, Guangyuan Liu, Xiting Huang

**Affiliations:** ^1^Post-doctoral Station of Mathematics, Southwest University, Chongqing, China; ^2^Faculty of Psychology, Southwest University, Chongqing, China; ^3^Department of Education, Chongqing University of Arts and Sciences, Chongqing, China; ^4^School of Electronic and Information Engineering, Southwest University, Chongqing, China

**Keywords:** sex differences, oxytocin, working memory, facial expressions, identity

## Abstract

Although oxytocin (OXT) has been shown to increase the ability of face perception and processing, no study has explored whether it could improve the performance of working memory for emotional expression information in males and females. Thus, we performed a double-blind, mixed-design, placebo-controlled study to investigate the effects of OXT on temporary maintenance/manipulation of facial information through a facial expression (EMO) vs. identity (ID) working memory task, both for males (*N* = 45) and females (*N* = 46). Our results showed that in female participants, OXT increased the accuracy of the recognition of faces displaying angry and happy emotions, in the EMO tasks, and also reduced the response time to negative emotional faces, in the ID task. However, the above effects were not present in male subjects. These results indicate that OXT may increase the efficiency of working memory in face processing and this trend is reflected in females rather than in males. This study provides novel evidence for the sexually dimorphic effects of OXT on social cognition.

## Introduction

Human faces convey both changeable (e.g., emotional expressions) and invariant (e.g., identity) facial features (Haxby et al., 2000). The emotional expressions can communicate a person's emotional internal state, while the identity enables us to link current events with individual, specific, prior experience. Yet, it is critical to keep track of someone's identity and their emotional states over time so that people can plan appropriate behavioral responses. This suggests that temporary, or working memory for emotional expression and identity information may play an important role in the social interaction progress (Jackson et al., 2014).

In the laboratory, the n-back task is used as a common experimental paradigm to explore the working memory for facial information. During the task, participants are asked to focus on either the emotion (EMO) or the identity (ID) in a series of emotional faces, and respond whether the currently presented emotional face is the same as the one presented in previous trials. Through this experimental paradigm, researchers have made a series of interesting discoveries. For the EMO task, Xin and Lei (2015) found that there was a significant positive correlation between the response accuracy and empathy scores. They explained that participants with higher empathic ability are inclined to pay more attention to facial emotional features and ignore irrelevant information, thus they can encode face emotional information better. As for the ID task, maybe because negative-related stimuli can capture more attention than neutral or positive stimuli, several studies reported that negative emotional expression could facilitate the participants' face identity working memory (Jackson et al., 2008, 2009; Sessa et al., 2011). In summary, people who can pay closer attention to facial features may find it easier to encode relevant information into a mental representation, so that they have better performance in the working memory for facial information.

Oxytocin (OXT)'s effects on social cognition of human beings have become a major focus in recent years (Meyer-Lindenberg et al., 2011; Kumsta and Heinrichs, 2013). Intranasal dosing of OXT, which is believed to cross the blood–brain barrier and achieve access to the central nervous system (CNS) (Neumann et al., 2013; Striepens et al., 2013), has been found to increase the efficiency of face perception and processing (Van IJzendoorn and Bakermans-Kranenburg, 2012). Many studies have found that OXT administration can enhance emotion recognition performance (Lischke et al., 2012; Leknes et al., 2013; Shahrestani et al., 2013), and a further study showed that the enhancement of the performance is concomitant with increased recruitment of attentional resources (Prehn et al., 2013). According to the social adaption model (SAM) proposed by Ma et al. (2016), OXT is an evolutionarily-conserved neuropeptide hormone; its main social adaptation functions including regulation of negative affect, and promotion of social motivation to initiate and maintain social interactions. Considering previous effects of OXT on face perception and processing, we asked whether OXT administration may improve the performance of working memory for emotional expression information. Thus, we investigated this point, which, to the best of our knowledge, has not yet been explored.

It should also be noted that, the sexually dimorphic effects of OXT on social cognition cannot be ignored in current studies. Many previous research, including studies on negative emotional face processing, social approach/avoidance tendency (Theodoridou et al., 2013; Preckel et al., 2014), and social cooperation/competition (Fischer-Shofty et al., 2013; Scheele et al., 2014), have found inconsistent or even opposite results of the OXT's effects in males and females. However, there are also some studies that do not support the OXT's sexual dimorphism, such as on approach-avoidance tendency to emotional facial stimuli (Theodoridou et al., 2013) and the dampening of basic physiological arousal (Ellenbogen et al., 2014). Because of this, in our exploration of the OXT effects on working memory of emotional expression information, we also wondered whether there exist differences between males and females, and this is the second question we wanted to investigate.

In conclusion, we drew on a facial EMO and ID working memory task in this double-blind, placebo-controlled, mixed design study to explore the impact of OXT on the temporary maintenance/manipulation of facial information, both in male and female participants. Our overall hypothesis is that OXT administration could enhance performance on both the EMO and ID working memory task, and sex may play a potential regulatory role in this effect.

## Methods

### Participants and treatment

We recruited 87 undergraduate and graduate students via advertisements posted in the Bulletin Board System of both the Southwest University and the Chongqing University of Arts and Sciences. Each subject was given written description about the study and then provided their informed and written consent, prior to participation. Not one of the subjects was taking any form of medication, or reported suffering from neurological problems or psychiatric illness before the study. No female subject was menstruating (Bakermans-Kranenburg and van Ijzendoorn, 2013), and no one was either using oral contraceptives or pregnant. In addition, at least 12 h before the experiment, subjects were asked to maintain a regular sleep pattern and abstain from smoking, alcohol, and caffeine. The Ethics Committee of Southwest University and Chongqing University of Arts and Sciences approved to carry out this study, with all of the involving procedures, which conformed with the sixth revision of the Declaration of Helsinki.

**Figure 1:**
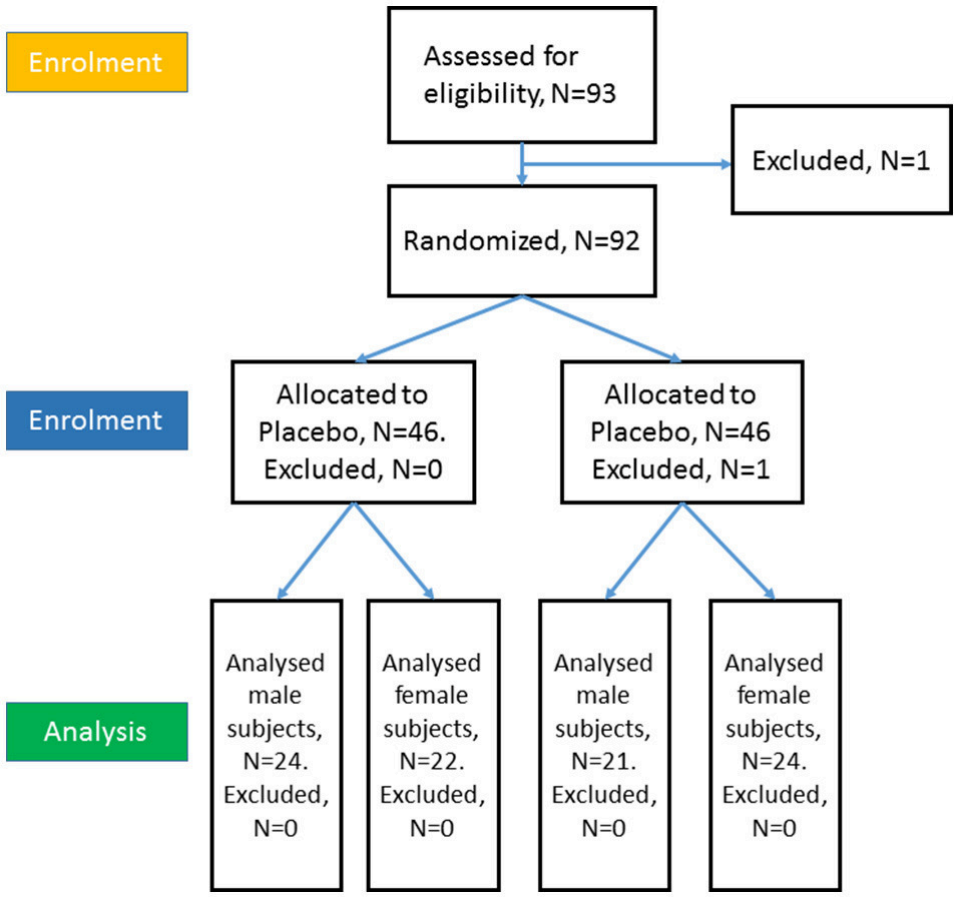
CONSORT flow diagram of the participants.

An earlier study on exploring the sex differences of OXT used a sample sizes of 74 (Gao et al., 2016 with [partial] eta-squared of 0.09). We relied on the effect size to estimate the required sample size for our study, using the above eta-squared as inputs in G-Power 3.1 (Faul et al., 2007) with a power of 0.8 at an alpha of 0.05, yielded a required sample size of 82, with at least 21 participants per group. Thus, we recruited 93 healthy students (46 males, 47 females, with a mean age of 21.2 years; S.D. = 1.76) to participate in our study. Among them, a male withdrew from a cold at the stage of drug application, and a female was excluded because she did not reach the correct level in the practice stage. 24 females and 21 males were treated with OXT, with the remaining 46 subjects receiving the PLC (placebo) treatment, which met the requirements of the number of participants in each group (see Figure 1). Before the experiment, all subjects were first administered a single intra-nasal dose of 24IU OXT (Oxytocin Spray, Sichuan Meike Pharmacy Co. Ltd, China; three puffs of 4IU per nostril with 30 s between each puff) or PLC (containing all of the same ingredients as the Oxytocin Spray except for the neuropeptide, (also three puffs per nostril). After 45 min of the OXT or PLC treatments, the formal experiment started, which was conducted in line with the study of Striepens et al. (2011).

### Experimental design

To control for the potential confounding effects of OXT on empathy, which may interfere participants' working memory performance for facial information (Xin and Lei, 2015), all subjects completed an Inter-personal Reactivity Index (IRI)-C (Zhang et al., 2010) immediately prior to the experiment. This index is one of the most effective instruments for measuring trait empathy. The IRI-C contains 22 items, could be divided across four sub-scales: (i) the perspective taking (PT) sub-scale measures the tendency to spontaneously adopt the point of view of others; (ii) the fantasy(FS) scale assesses the tendency to transpose oneself imaginatively into the feelings of fictitious characters; (iii) the empathic concern (EC) scale measures an “other-oriented” compassion or concern for unfortunate others; and (iv) the personal distress (PD) scale assesses a “self-oriented” reaction to others' negative emotions.

**Figure 2:**
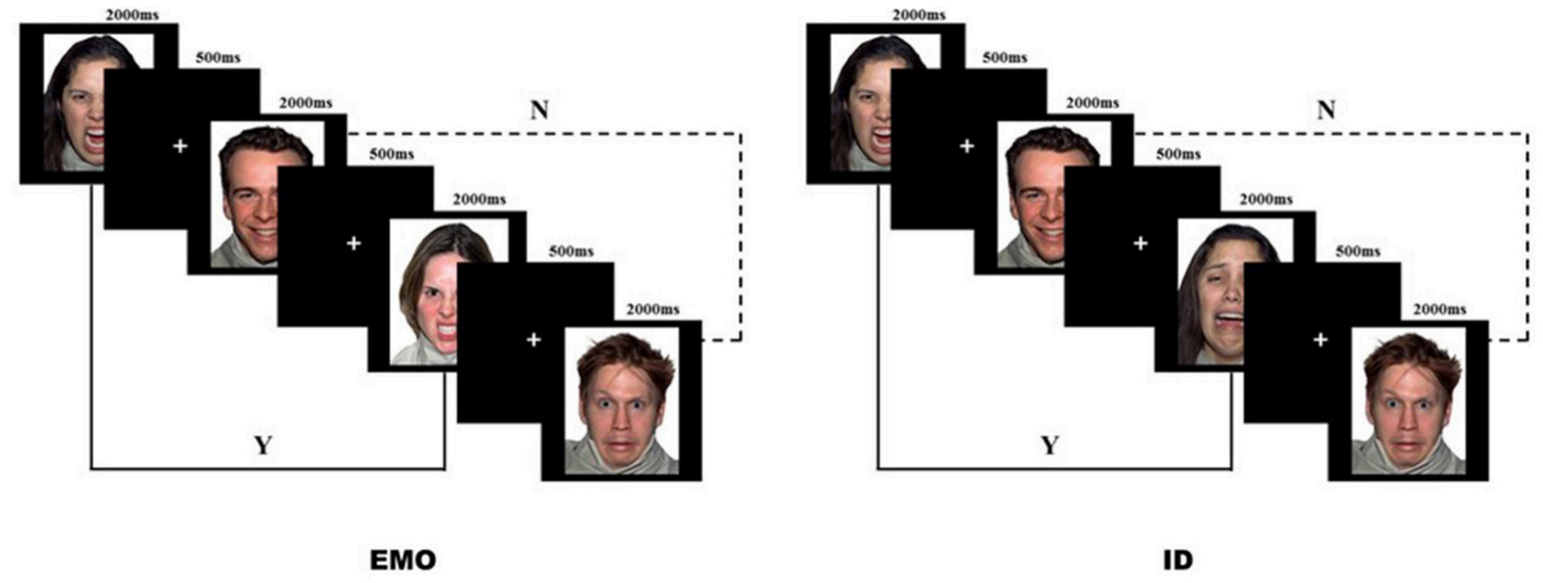
Facial Expression vs. Identity 2-back task. Angry, happy, fearful, and sad emotional faces (as acted by two females and two males) were displayed for 2,000 ms, followed by a fixation cross for 500 ms. Participants were instructed to respond if the current stimulus matched (Y) or mismatched (N) the stimulus presented two trials prior to the current stimulus.

Sixteen face images (two females and two males displaying angry, happy, fearful, and sad expressions) from the NimStim standardized facial expression stimulus set (Tottenham et al., 2009) were chosen for the task stimuli. Participants performed an emotional face working memory task run, based on the 2-back paradigm (Figure 2). In the experiments, they were instructed to match faces according to only one aspect (EMO or ID) of the face stimuli and ignore the other (Neta and Whalen, 2011). For example, during the EMO task, the subjects were asked to press the “F” button if the current EMO of the face matched the EMO presented two trials earlier, and the “J” button if different. The order of task was counter-balanced across participants, thus half of the participants carried out the EMO task first, and the other half carried out the ID task first.

Before the formal experiment began, the subjects were required to practice in order to ensure that they fully understood the requirements of the two tasks. Only after achieving a correct score of more than 60% in the practice, were subjects allowed to start the formal experiment (one female participant was excluded for not reaching the above standard). During the formal experiment, either the word “emotion” or “identity” would appear to remind the participants which task they would perform. Each task included four blocks, with 32 faces presented in a pseudo-random sequence. Each face stimulus was displayed for 2,000 ms, followed by a 500 ms cross-fixation. At the end of each block, the subjects were allowed to rest. The next block was started once the subjects confirmed that they had rested.

The results were analyzed via SPSS 16.0. Greenhouse-Geisser corrections were used when the sphericity hypothesis was violated, and Bonferroni corrections were applied when follow-up tests were required.

## Results

### Empathy scores analysis

The ANOVA analysis pertaining to the sex (male vs. female) × drug (OXT vs. PLC), according to the scores on the IRI-C showed that, regardless of the four sub-dimensions or the total, there were no significant main effects of sex and drug, and there was no treatment × sex interaction, either (all *ps* > 0.12) (Table 1).

**Table 1:** Empathy scores for male and female subjects in OXT and PLC groups.

	**OXT**		**PLC**	
	**Male**	**Female**	**Male**	**Female**
EC	3.66 ± 0.52	3.72 ± 0.47	3.71 ± 0.47	3.58 ± 0.40
FS	3.53 ± 0.56	3.70 ± 0.65	3.65 ± 0.45	3.46 ± 0.56
PD	2.87 ± 0.72	3.04 ± 0.73	2.90 ± 0.67	3.19 ± 0.63
PT	3.60 ± 0.60	3.86 ± 0.35	3.60 ± 0.58	3.53 ± 0.48
Total	3.41 ± 0.34	3.58 ± 0.33	3.46 ± 0.29	3.44 ± 0.29

### Accuracy analysis

**Figure 3:**
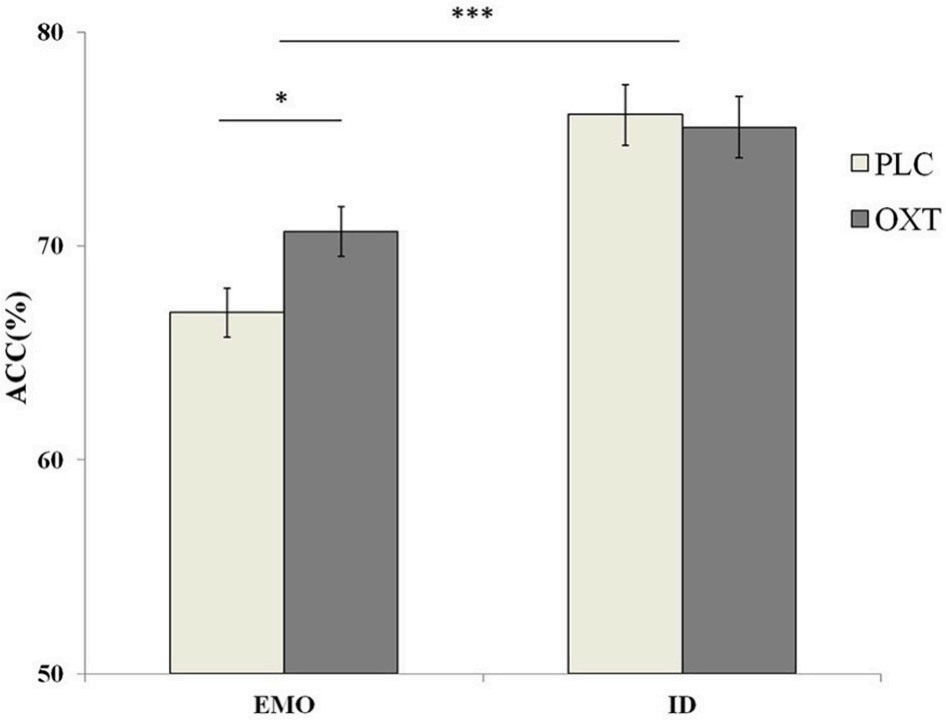
Behavioral performance (mean ± standard error) during the EMO and ID tasks. Participants were significantly less accurate with the EMO task than the ID task. The OXT group was more accurate with the EMO task than the OXT group. The symbol ^*^ indicates significant differences at *p* < 0.05, and ^***^ indicates significant differences at *p* < 0.001.

A repeated-measures analysis of variance (ANOVA), on the accuracy with treatment type (OXT vs. PLC), sex (male vs. female) as between-subjects' factors and task (EMO vs. ID), and emotional types (sad vs. fearful vs. angry vs. happy) as within-subjects' factors, revealed a significant main effect of task type [*F*_(1, 87)_ = 49.05, *p* < 0.001, η^2^_*p*_ = 0.36]. Participants were significantly more accurate in the ID task (mean ± S.E.: 75.85 ± 1.00%), than they were during the EMO task (mean ± S.E.: 68.77 ± 0.80%) (Figure 3). These results were consistent with previous research findings (Neta and Whalen, 2011; Xin and Lei, 2015), indicating the increased cognitive load involved in EMO recognition when compared with identity recognition. Another significant main effect of the emotional task [*F*_(3, 261)_ = 12.83, *p* < 0.001, η^2^_*p*_ = 0.31] was discovered. *Post-hoc* paired comparisons showed that the ACC for the happy faces (M = 75.67, S.E. = 0.83) were significantly higher than those for the other three emotional faces (sad, fearful, and angry, respectively) (mean ± S.E.: 71.48 ± 0.92%, 71.53 ± 0.89%, and 70.56 ± 1.03%, *p* < 0.001 in each case).

**Figure 4:**
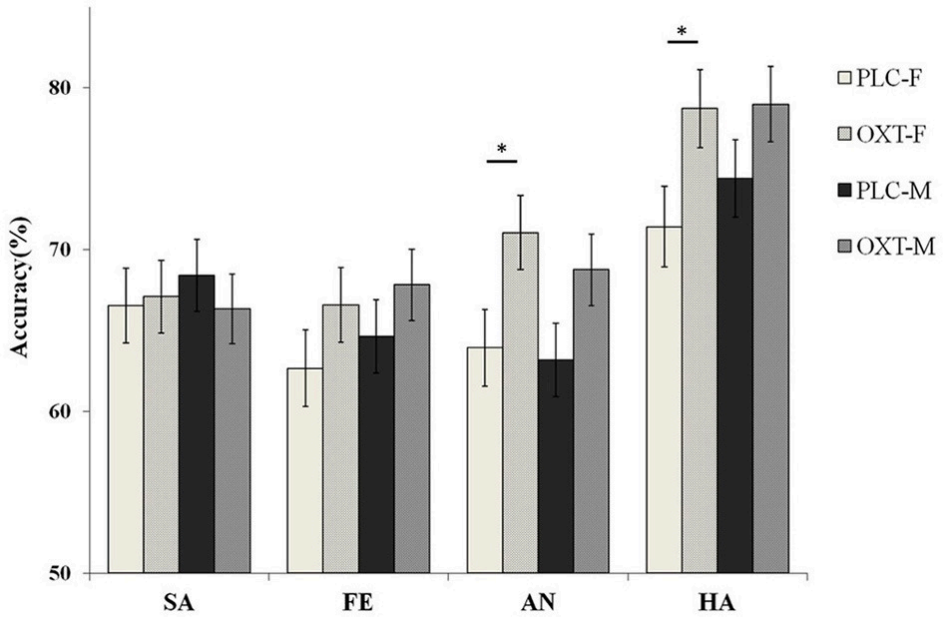
Gender differences in terms of behavioral performance (mean ± standard error) on the EMO task. Female(F) participants in the OXT group showed increased ACC with the angry(AN) and happy (HA) emotional faces as compared with the PLC group. Male(M) subjects displayed no significant effects in their performance after OXT treatment. Here, ^*^ indicates significant differences at *p* < 0.05.

Although we did not find the significant main effect of treatment, the interaction between treatment, task, and emotional type was significant [*F*_(3, 261)_ = 2.81, *p* < 0.05, η^2^_*p*_ = 0.09]. Our results show that the participants, in the OXT group only, increased the accuracy of the angry [*F*_(1, 87)_ = 7.69, *p* < 0.01, η^2^_*p*_ = 0.08] and happy [*F*_(1, 87)_ = 7.14, *p* < 0.01, η^2^_*p*_ = 0.08] emotional faces in the EMO task, but no effect was noted with the sad and fearful faces. No OXT affect was observed during the ID task. To examine any potential differences, in terms of the effects of OXT on different genders, in the EMO task, we analyzed the data separately for male and female participants. Our results indicated that female participants, in the OXT group, showed increased accuracy with the angry and happy emotional faces, compared with the PLC group [63.93 ± 2.22 vs. 71.04 ± 2.27%, *F*_(1, 87)_ = 5.00, *p* < 0.05, η^2^ = 0.05 and 71.41 ± 2.22 vs. 78.71 ± 2.21%, *F*_(1, 87)_ = 5.57, *p* < 0.05, η^2^_*p*_ = 0.06]. For male subjects, no significant effects from OXT treatment were observed, on any of the four emotional tasks, with all *ps* > 0.22 (Figure 4).

### RT analysis

**Figure 5:**
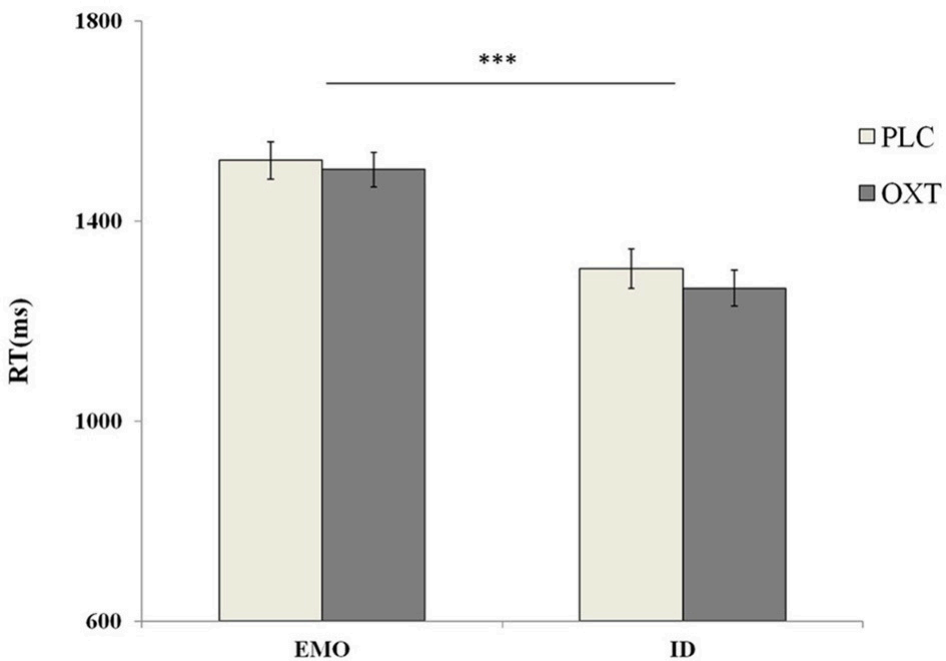
Behavioral performance (mean ± standard error) during the EMO and ID tasks. Participants were significantly slower to respond during the EMO task than the ID task. Here, ^***^ indicates significant differences at *p* < 0.001.

A repeated-measures analysis of variance (ANOVA), of accuracy with treatment type (OXT vs. PLC), sex (male vs. female) as between-subjects' factors and tasks (EMO vs. ID), emotional types (sad vs. fearful vs. angry vs. happy) as within-subjects' factors, revealed a significant main effect in terms of task type [*F*_(1, 87)_ = 82.56, *p* < 0.001, η^2^_*p*_ = 0.49]. Participants were significantly quicker to respond during the ID task (mean ± S.E.: 1284.51 ± 24.69 ms), than during the EMO task (mean ± S.E.: 1511.66 ± 27.13 ms) (Figure 5). These results were also consistent with previous research findings (Neta and Whalen, 2011; Xin and Lei, 2015). No other significant main effects (such as treatment, gender, and emotional types) were noted.

**Figure 6:**
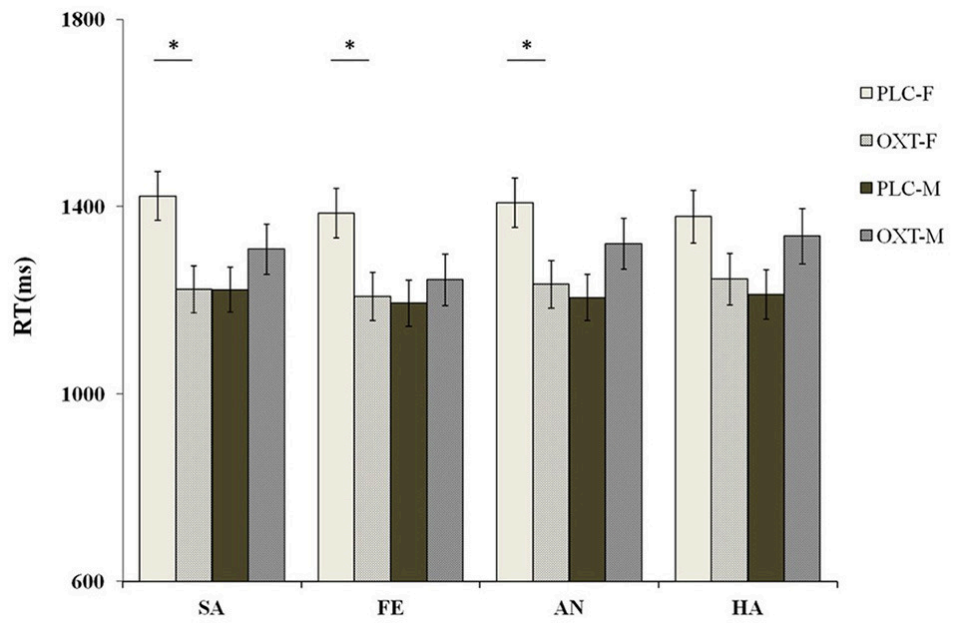
Sex differences of the behavioral performance (mean ± standard error) during the ID task. OXT accelerated the female participants' reaction response time to the sad (SA), fearful (FA), and angry (AN) faces, as compared with the PLC group in the ID task. However, no OXT affect was observed in the male participants. Here, ^*^ indicates significant differences at *p* < 0.05.

We also found evidence of a significant interaction between treatment, task, and sex [*F*_(1, 87)_ = 5.96, *p* < 0.05, η^2^_*p*_ = 0.07]. The exploratory simple effects test showed that, compared with the PLC group, females in the OXT group finished the ID task quicker than males in the OXT group (1399.07 ± 47.70 vs. 1208.61 ± 48.72 ms). However, no such effects were observed during the EMO task. To clarify potential gender differences, in terms of the emotional task, we carried out a similar separate analysis of male and female participants. For the female participants, when compared with the PLC group, OXT accelerated the reaction time to the sad [*F*_(1, 87)_ = 7.18, *p* < 0.01, η^2^_*p*_ = 0.08], fearful [*F*_(1, 87)_ = 6.27, *p* < 0.05, η^2^_*p*_ = 0.07], and angry [*F*_(1, 87)_ = 6.53, *p* < 0.05, η^2^_*p*_ = 0.05] faces, during the ID task, at a significant level. Even with the happy faces, a marginally significant level (*p* = 0.09) was observed. However, as observed, OXT did not accelerate the speed of the response of male subjects to ID tasks, during any of the four emotional tasks (all *ps* > 0.11) (Figure 6).

## Discussion

In this study, we used a facial EMO and ID recognition working memory task to explore whether or not OXT affect the temporary maintenance/manipulation of facial information and the potential differences between males and females. Our results showed that OXT enhanced the accuracy of the recognition of angry and happy faces in the facial EMO recognition working memory task. In addition, OXT accelerated the reaction time in females' negative facial identity (such as sad, angry, and fearful faces) on the recognition working memory task. However, no significant OXT effects were observed in male participants. Combining the results of previous studies, our discussion is as follows.

In the EMO task, we found that OXT administration improved the accuracy of angry and happy faces in female participants, but not in sad and fearful faces. According to Xin and Lei (2015), individuals who are able to focus their attention more (also characterized by a higher level of empathy), on facial EMO perception, could reallocate cognitive resources from task-irrelevant processes to task-relevant processes, which would further improve their accuracy. While previous studies indicated that OXT can increase recruitment of attentional resources to facial cues (Gamer et al., 2010; Tollenaar et al., 2013), we could infer that, because of the effects of OXT, participants could pay more attention to the facial EMO information and ignore the interference of irrelevant information, compared with the PLC group. Perhaps because of this, the participants with intranasal OXT had more facial EMO information encoded into a mental representation, which may have made them have higher accuracy than the PLC group. As to the limited influence of OXT on the angry and happy faces, we suspect that it may be due to the adaptive function of OXT. According to previous studies relating to motivational direction, anger, and happiness are both approach-related social emotions. While Preckel et al. (2014) reported that OXT in women facilitates approach behavior in a real-life setting with other people, the “tend-and-befriend” tendencies caused by OXT may have made our female participants more sensitive to angry and happy faces, thus increasing their attention level.

Our results also showed that OXT could accelerate females' reaction times during the negative facial identity recognition working memory task. Neta and Whalen (2011) reported that the activity of fusiform gyrus, which is related to the visual processing of the face (Kanwisher and Yovel, 2006), could predict faster performance of the subjects. While previous researches had reported that the activity of fusiform gyrus could be modulated after the administration of OXT (Domes et al., 2007; Kanat et al., 2015), we may conclude that OXT can make women more aware of, and concerned about, other people's facial features, thus accelerating their response time in the ID task. However, the accelerating effect of OXT on reaction time is only reflected in negative emotional faces of the female participants. Unlike the EMO recognition task, the identity recognition task only required participants to process non-variant facial structure features, and emotion was merely an implicit element. Therefore, it is reasonable to believe that the accelerating effect is due to the interaction between OXT and emotional valence. Indeed, previous studies have shown that OXT could make female participants highly vigilant against threatening emotional stimuli, to exhibit defensive behavior (which is contrary to the conclusion previously drawn from male subjects) (Domes et al., 2010; Wittfoth-Schardt et al., 2012; Gao et al., 2016), and negatively-valenced faces could receive a greater proportion of resources per item than non-threatening or neutral faces, which may enable a timely response in a working memory task for face identities.

It is noteworthy that, regardless of whether participants were performing the EMO or ID tasks, OXT only affected the female subjects, but no significant effect on male subjects was observed. Thus, our results supported many previous studies, which showed that intranasal OXT could produce sex-dependent effects on human cognition. Combined with the results of previous studies, the differences in OXT receptor affinity offer one possible explanation for the sexually-dimorphic results of our study. Some research showed that gonadal steroid hormones, such as estradiol and progesterone, could modulate the OXT receptor (Gimpl and Fahrenholz, 2002; Choleris et al., 2008). Thus, the existing differences between both sexes in the sensitivity of the OXT system when administration with OXT may be attributed to different gonadal steroid hormones (Hawkins and Matzuk, 2008) in women and men. In addition, the interaction of baseline and post-drug OXT levels may also provide a potential alternative explanation. According to the results of Altemus et al. (1999), the baseline OXT levels of females in cerebrospinal fluid (CSF) are higher than those of males. Thus, raising brain OXT levels in females through drug administration may come closer to the maximum level of its effect on working memory in face processing compared to males. This hypothesis could be evaluated by measuring dose–response properties of intranasal OXT and the baseline CSF of OXT in future experiments.

Due to the OXT's effects on promoting social adaptation in healthy and clinical populations, few researchers suggested that the potential therapeutic applications of OXT are promising (Watanabe et al., 2015; Ma et al., 2016). Indeed, using OXT in the field of clinical practice is a possibility worth addressing in future research. However, the sex differences of the resulting OXT effect on facial expression and identity working memory that were found in the current study remind us, that extensive research is essential to inspect the potentially sex-dependent effects of OXT prior to widespread clinical application.

## Limitations

This study has several limitations, future studies may need to improve. First, this study was not pre-registered, which is generally recommended for future research in this area. Then, the modulatory role of gonadal steroids on the OXT's effects should be investigated in future studies, e.g., by examining the hormonal change of women in different cycle phases in comparison to men. Ultimately, the behavioral indicators (response time and accuracy) we choose in our study may be less sensitive to the effects of OXT; consequently, future researchers could select other indicators (e.g., event-related potentials studies) to further verify and explore this topic.

## Conclusion

In this study, we explored the effects of OXT on the temporary maintenance/manipulation of facial information in men and women, through a facial expression vs. identity working memory task. Our results showed that, in female participants, OXT increased the accuracy of the recognition of faces displaying angry and happy emotions in the EMO tasks, and also reduced the response time to negative emotional faces in the ID task. However, the above effects were not present in male subjects. These results indicate that OXT may increase the ability of working memory for face processing and this trend is mainly reflected in females but not in males.

## Author contributions

TY, GL, and XH designed experiments. TY and CY carried out experiments. TY analyzed sequencing data and wrote the manuscript.

### Conflict of interest statement

The authors declare that the research was conducted in the absence of any commercial or financial relationships that could be construed as a potential conflict of interest.
